# Optical Fibre Sensor for Simultaneous Measurement of Capillary Refill Time and Contact Pressure

**DOI:** 10.3390/s20051388

**Published:** 2020-03-03

**Authors:** Chong Liu, Ricardo Correia, Hattan Ballaji, Serhiy Korposh, Barrie Hayes-Gill, Stephen Morgan

**Affiliations:** Optics and Photonics Group, Faculty of Engineering, University of Nottingham, University Park, Nottingham NG7 2RD, UK; chong.liu@nottingham.ac.uk (C.L.); Ricardo.GoncalvesCorreia@nottingham.ac.uk (R.C.); S.Korposh@nottingham.ac.uk (S.K.);

**Keywords:** capillary refill time, plastic optical fibre, photoplethysmography, fibre bragg grating, contact pressure

## Abstract

The widely applied capillary refill time (CRT) measurement is currently performed by manually applying pressure and using a stopwatch to record the time taken for the skin to recover its normal colour after a blanching pressure is applied. This method is highly subjective and observer-dependent. This paper presents a new, integrated optical sensor probe, combining monitoring of the capillary refilling process with the blanching pressure applied. The sensor consists of an optical fibre-based reflectance photoplethysmography (PPG) sensor to measure the reflected light signal, as well as a fibre Bragg grating (FBG) to measure the applied blanching pressure and to indicate the time when pressure is released. This sensor was applied to calculate the CRT (1.38 ± 0.66 s) of 10 healthy adult volunteers with (55.2 ± 21.8 kPa) blanching pressures. The form of the capillary refilling data was investigated by fitting using an exponential regression model (R^2^ > 0.96). The integrated probe has the potential to improve the reliability of CRT measurements by standardising the optimum duration and magnitude of the pressure.

## 1. Introduction

Capillary refill time (CRT) is defined as the time taken for a distal capillary bed to regain its colour after blanching caused by external applied pressure. This test is widely applied in clinical care as a simple means of cardiovascular assessment, and has been proposed as a method for the clinical assessment of peripheral macrovascular disease and cutaneous microvascular disease [1]. CRT was first introduced in 1910 as an indicator of dehydration [2], and since the 1940s, it has been accepted as a medical indicator of dehydration and surgical shock [3,4]. CRT has also been applied in the assessment of perfusion [5], and there is interest in areas such as assessment of tissue breakdown leading to foot ulceration for people with diabetes [1,6,7].

Currently, there is no clearly defined and standardised method for CRT measurement [8]. The commonly applied method of measuring CRT is using a stopwatch to manually record the time taken for the skin to recover its colour after blanching [9,10]. The testing area is usually set close to the finger or toenail [11]. As the predominant method of calculating CRT is highly observer-dependent and influenced by skin temperature, with a lack of standardisation of the actual blanching manoeuvre (e.g., pressure strength: light, moderate, or firm; pressure duration: 3 s, 5 s, or until the capillary bed visually blanches), the obtained results are likely to be unreliable and inaccurate [12,13,14,15,16].

Recent studies investigating automatic measurement of this process have reported that CRT can be calculated from the changes in the reflected light due to the pressure applied on the skin [13,17,18,19,20,21]. As the reflected photoplethysmogram depends on the change of blood volume, it can be used to calculate the CRT. In previous studies [10,22], CRT sensors were designed based on a reflectance PPG sensor, which was simply composed of a light source (LED) and a photodetector. This sensor design enables CRT measurement on any part of the subject’s body, as it is based on reflectance geometry [23]. However, these designs did not take the blanching pressure into consideration, which is a limitation as the duration and strength of the blanching pressure can influence the measured CRT [10,24]. Therefore, simultaneous recording of the applied pressure and PPG can be beneficial in classifying normal and abnormal CRT. In a previous study, we proposed the incorporation of polymer optical fibre (POF) pulse oximetry with a fibre Bragg grating (FBG) pressure sensor to simultaneously monitor oxygen saturation and the contact force of the subject [25]. Compared to other optical fibre-based pressure sensors, such as the extrinsic Fabry-Perot interferometry, the FBG-based pressure sensor is more suitable for skin contact measurements as no additional diaphragm is required, making it more robust [26,27,28]. Although a simple approach, POF displacement sensors do not provide the sensitivity of FBG pressure sensors [29,30]; therefore, FBG contact pressure sensing is preferred. Thus, in the present study, this sensor is used to simultaneously monitor the reflectance PPG signals and the applied pressure signals to provide a new method of measuring CRT. In this study, the pressure recording is utilised in signal processing to indicate the onset and release of the applied pressure.

After obtaining the reflected PPG signal, the CRT can be calculated from the processed refilling signals. An exponential fitting model of the light intensity data of the recovery phase has been applied for CRT calculation [8,31]. In 2017, Shinozaki et al. presented a CRT measurement method which modelled the recovery phase of the intensity waveform as an exponential decay, and recorded the time taken for the light intensity to return to 10% of its initial height above baseline after the blanching pressure was released [31]. However, the relationship between the order of exponential regression model and the calculated CRT was not investigated in detail. In order to try to better understand the CRT process, this paper further investigates and demonstrates the relationship between the order of exponential models and the calculated CRT.

This paper demonstrates the application of a novel optical fibre sensor probe to detect the reflectance PPG signal and the blanching pressure in order to measure the CRT. The next section describes the design of the sensing system. Calibration and in vivo experiment results are shown in Section 3, and Section 4 presents the conclusion.

## 2. Materials and Methodology

### 2.1. CRT Monitoring System

**Figure 1:**
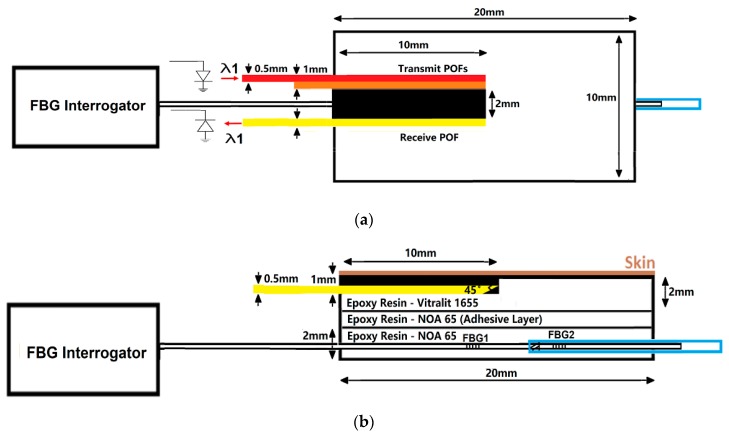
(**a**) Schematic diagram of a plane view of the probe. The red frame represents the POF connected to an infrared LED (centre wavelength λ = 850 nm). The yellow frame represents the POF connected to a photodiode. As the sensor has been adapted from a pulse oximeter described previously, there is an additional channel (orange frame) that exists which was not used in this investigation. The blue frame represents the stainless-steel tube. (**b**) Schematic diagram of a side view of the probe. The two sensors are encased in epoxy. The POFs transfer light from/to the skin, whilst the FBGs monitor the applied pressure. POF – polymer optical fibre and FBG – fibre Bragg grating, LED - Light emitting diode.

**Figure 2:**
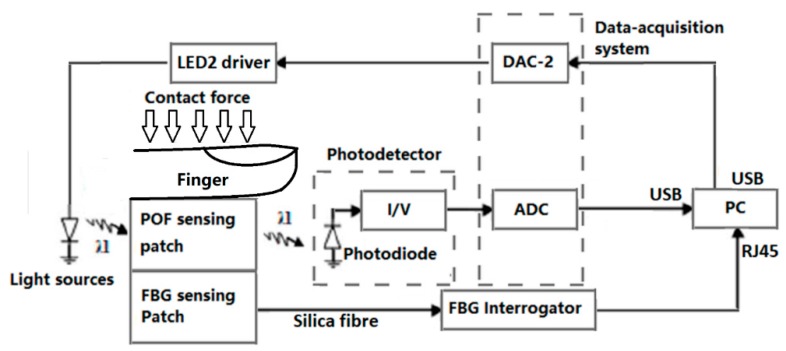
Opto-electronics system for the designed probe. An infrared fibre-coupled LED (λ = 850 nm, Thorlabs) is applied as the light source. The photo detector is a Si-type photodiode (PDA36A-EC, Thorlabs gain = 4.75 × 10^6^ Ω). A NI-myDAQ device (National Instruments, Austin, TX, USA) was applied, as the data acquisition system in the opto-electronics system had a +/−10 V 16-bit analogue-to-digital converter (ADC). The FBG interrogator is a SmartScope FBG interrogator (0.8 pm resolution). (I/V: current-to-voltage converter; DAC: digital-to-analogue converter; ADC: analogue-to-digital converter; and PC: personal computer).

Figure 1 shows the design of the integrated probe, which combines a POF perfusion sensor and an FBG pressure sensor. The perfusion sensor combines three 500 µm diameter POFs to transfer light from/to the opto-electronic system (shown in Figure 2). These fibres are encased in a bio-compatible epoxy (Vitralit 1655) which directly comes into contact with the skin during measurement. The end of each fibre has been cleaved to 45 degrees and polished in order to increase the light coupling efficiency transmitted to, and received from, the skin [32]. An absorbing black rubber shield (2 mm thick) was situated between the transmitting and receiving optical fibres in order to prevent light from passing directly between the source to the detector, which would cause a large DC (direct current, i.e., a static, unmodulated signal) background light level. The large DC background light level would make it more difficult to isolate the CRT signal due to increased shot noise and would make a high-resolution analogue-to-digital converter be required.

The pressure sensor is a 125 µm diameter optical fibre containing two FBGs (separation = 6 mm) encased in an epoxy (Norland Optical Adhesive 65). The purpose of the epoxy is to transduce the transverse load applied to FBG1 into an axial strain, which can be measured using an optical interrogation unit. It has a Young’s modulus (137.9 MPa) lower than that of the optical fibre, which increases the strain induced on the FBG under a transverse load [33,34]. FBGs are also sensitive to temperature, and so a second FBG (FBG2) is used for temperature compensation [25]. The FBG2 is located inside a stainless-steel tube (internal diameter = 0.31 mm) in order to isolate it from the effects of pressure [35,36]. In this experiment, the integrated sensor probe was attached to the optical table in order to prevent manually straining the fibre, which would affect the FBG response.

Figure 2 presents the schematic of the opto-electronic system. The reflectance PPG monitoring system is composed of an infrared LED (centre wavelength λ = 850 nm, M850F2 fibre-coupled LED, Thorlabs, Newton, NJ, USA) and a photodiode (PDA36A-EC 350–1000 nm, Thorlabs, Newton, NJ, USA). This wavelength was selected as we adapted a pulse oximeter design; however, it should be noted that the green wavelength range is likely to provide higher contrast [10] and is likely to be adopted in future designs. The FBG interrogator (SmartScope, Smartfibres, Bracknell, UK) tracks the wavelengths reflected from the FBGs that indicates the applied pressure with temperature compensation.

### 2.2. FBG Calibration and Validation

The pressure sensor consists of two FBGs which are fabricated in the core of the same silica fibre (PS1250, Fibercore Ltd., Hampshire, UK). One FBG (FBG1) was covered by epoxy in order to increase the pressure sensitivity, whilst the other FBG (FBG2) was applied to compensate for the temperature effect on FBG1 [25].

Temperature calibration of the FBG sensors was achieved by placing the dual-FBG fibre in an oven (ED53, Binder GmbH, Tuttlingen, Germany). The oven increased the temperature around the FBG from approximately 30 °C to 49 °C, and the door was then opened to naturally decrease the temperature back to 30 °C. The temperature during the test was measured by a thermocouple (Type K, SE000 Pico Technology, Saint Neots, UK) with a resolution better than 0.025 °C and a range over −250 to +1370 °C. According to the temperature calibration result, as shown in the Supplementary Materials (Figure S1), the temperature compensation equation of FBG1 is:

(1) ΔλP= ΔλFBG1 − 2.4264×ΔλFBG2− 0.0111

where ΔλP is the Bragg wavelength shift in FBG1 with temperature compensation, and ΔλFBG1 and ΔλFBG2 are Bragg wavelength shifts read from the interrogator (all in pm).

**Figure 3:**
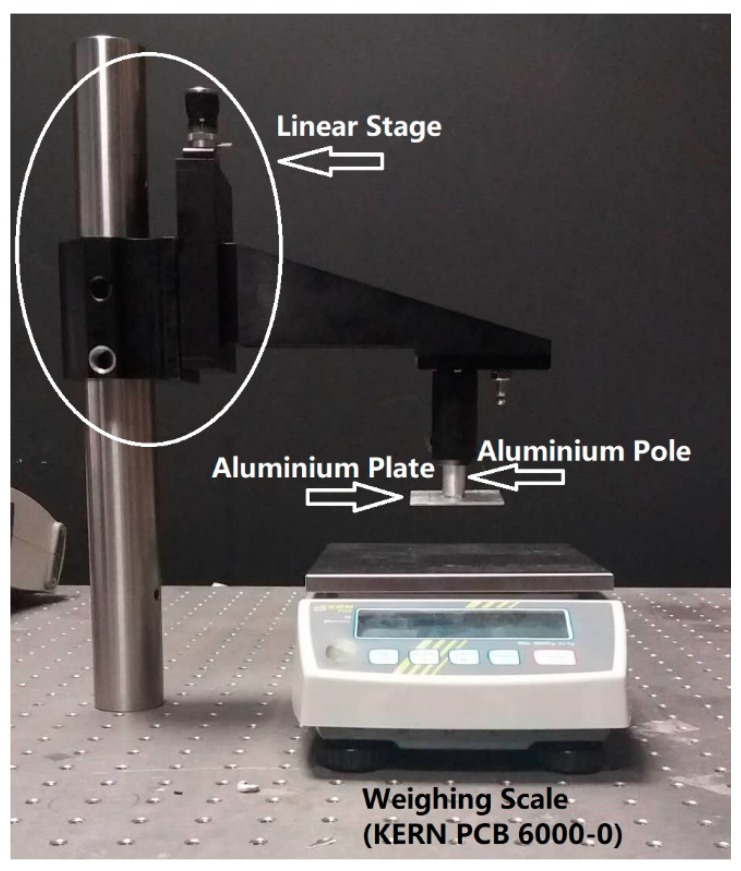
Pressure sensor calibration setup.

The pressure calibration of the sensor was performed using the setup illustrated in Figure 3. By moving a linear stage, the load was applied to the sensor through an aluminium pole and plate. The load applied during the test was recorded by a weighing scale (PCB 6000-0, KERN & SOHN GmbH, Balingen, Germany) that sat beneath the sensor and worked as a load cell. The FBG sensor’s sensitivity to pressure with the temperature compensation is:

(2) Pressure (kPa)=0.1563× (ΔλFBG1 − 2.4264 × ΔλFBG2 − 0.0111) – 0.0066.

### 2.3. Signal Processing

**Figure 4:**
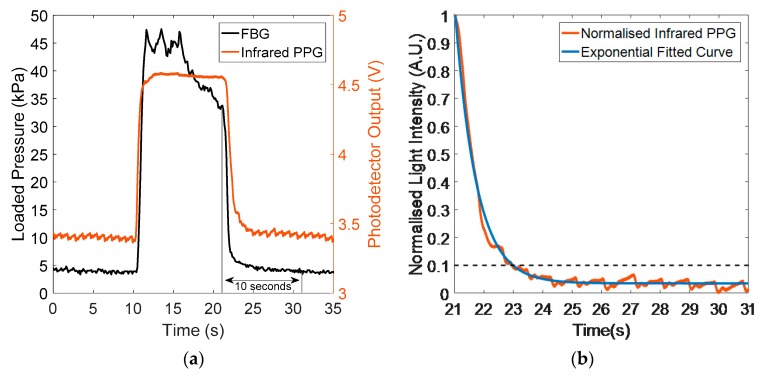
(**a**) Intensity changes (red curve) with loaded pressure (black curve). From 0 to 10 s, there is no external pressure applied on the finger, and both pressure sensor output and the reflected PPG are relatively stable. Then the light intensity rapidly increases in accordance with the pressure changes that arise from the blanching pressure (from 10.5 to 11.7 s). After the pressure is removed (at 21 s), the output pressure sensor rapidly returns to the baseline, whilst the reflected light intensity takes a longer time (CRT) to return to baseline. (**b**) Normalised reflected PPG signal (red curve) with exponential fitting curve (Equation (4), blue trace). The R-squared value of the exponential regression is 0.985, and the root mean square error (RMSE) is 0.024. The dotted line is the threshold applied for CRT estimation.

An exponential regression fit to the measured light intensity was used to calculate CRT [31], and is described here using an example. Figure 4a illustrates the intensity changes of the light reflected from the finger, with the loaded pressure recorded by the integrated PPG and contact pressure sensor. The recorded pressure data (black trace) can be processed with the reflected light signals (red trace) in order to find the optimum pressure for the CRT measurement. What’s more, the pressure recorded (black trace) can be used to isolate each capillary refill process during the 10 s following the point where the loading pressure is released. As the blanching pressure is manually applied by the subject, the pressure signal is not stable between 11.8 to 21.7 s.

From the results presented in Figure 4a, it can be observed that the DC component of the reflectance PPG signal is relatively stable after removing the load (apart from the oscillating heartbeat signal). Therefore, a horizontal linear fitting of the reflectance PPG DC component is used as the baseline. However, occasionally, motion artefacts, ambient light changes, and physiological changes will affect this baseline. In this case, normalisation of the extracted refilling curves is used to reduce the impact of changes in the baseline:

(3) $$I_{n}=\frac{\left(I_{max}-I_{min}\right)}{\left(I-I_{min}\right)}$$

where *I_n_* is the normalized light intensity of the reflected light signal, *I_max_* is the maximum reflected light intensity, *I_min_* is the minimum reflected light intensity, and I is the original reflected light intensity.

In Figure 4b, the red trace is the normalised isolated refilling curve, and the blue curve is the exponential fitting curve created according to the exponential fitting function:

(4) $$y=e^{A*I_{n}}+B_{0}$$

where $I_{n}$ is the normalized light intensity of the reflected light signal, A is the power of the exponential factor, and B_0_ is the baseline of the exponential fitting curve. As described in the introduction, the time taken for the light intensity to drop to a 10% threshold of the initial value above the baseline is the estimated CRT [10,31]. In Figure 4b, the isolated capillary refill process is represented by the exponential regression model, which demonstrates a high R-squared value (0.985). Consequently, the order (A) of the exponential regression curve can reflect the time of capillary refilling.

All PPG signal processing was carried out in MATLAB, version R2016a. The FBG signals are processed using the software SmartSoft V3.2.0 (Smart Fibres, Bracknell, UK). The Bragg peaks of two FBGs in the reflection spectrum are detected using the peak detection function of the SmartSoft V3.2.0.

### 2.4. In Vivo Capillary Refill Time Test Experiment

The designed POF sensor detects the intensity level of the reflected light from the skin which implies the tissue absorption changes with the applied load. The FBG sensor records the contact pressure between the skin and the probe, which indicates the time at which pressure is released and assists the CRT calculation.

**Figure 5:**
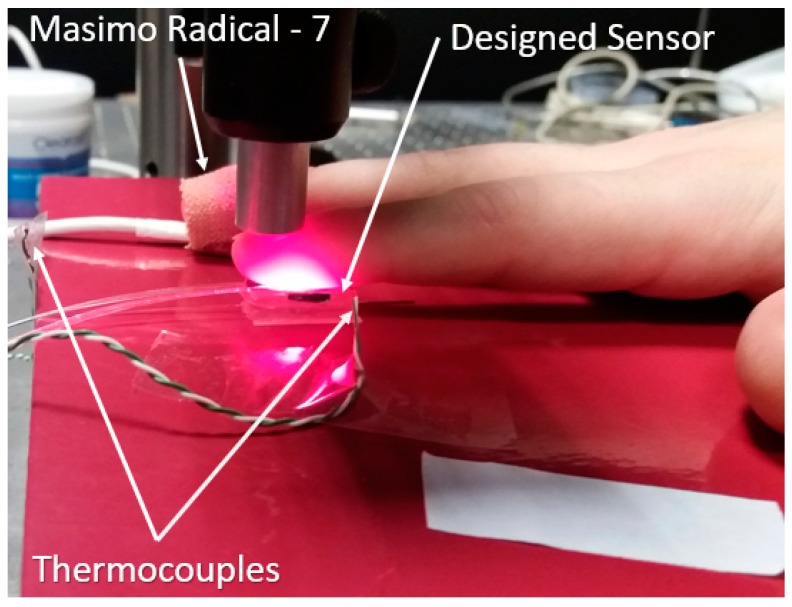
The region beneath the index finger is the designed optical fibre pressure sensor. The commercial sensor is taped onto the middle finger. Two thermocouples were used to measure the temperatures of the environment and the index finger.

Human volunteer studies were approved by the Faculty of Engineering Ethics Committee at the University of Nottingham. Figure 5 shows the experimental setup. Each volunteer was asked to locate their index finger on the sensor probe, and to press the probe manually by applying an external pressure which was high enough (over 15 kPa) to evacuate blood from the capillary bed, as no pulsatile PPG component from the reflected light could be observed [25].

As low body temperature would increase the CRT of the subjects [31,37,38], in this experiment, the laboratory temperature was maintained at a range from 24 to 26 °C to ensure that the skin temperature was higher than 30 °C (measured by a type K PICO Technology SE000 thermocouple (Pico Technology, Saint Neots, UK)).

## 3. Results and Discussion

### 3.1. Integrated Sensor Pressure Testing

**Figure 6:**
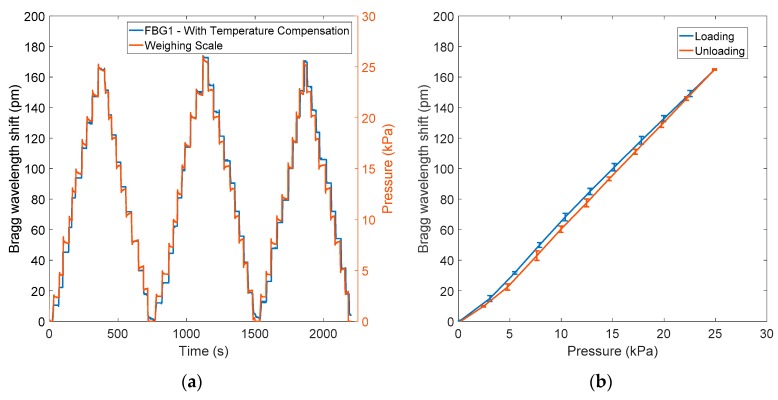
(**a**) Cyclical loading/unloading experiment up to 25 kPa. (**b**) Bragg wavelength shift versus contact pressure from Figure 6a, which demonstrates a linear relationship, repeatable results, and low hysteresis over three cycles.

Figure 6a shows the pressure response of the integrated sensor with temperature compensation, which follows that of the applied pressure. The applied pressure was increased/decreased by using the system shown in Figure 3. Figure 6b shows the dependence of the measured wavelength shift upon applied pressure during loading and unloading, demonstrating the capability of the integrated sensor to monitor pressure. Results demonstrating the effect of temperature on the FBGs and the effectiveness of the temperature reference calibration are shown in the Supplementary Material.

### 3.2. In Vivo Capillary Refill Time and Pressure Monitoring

**Figure 7:**
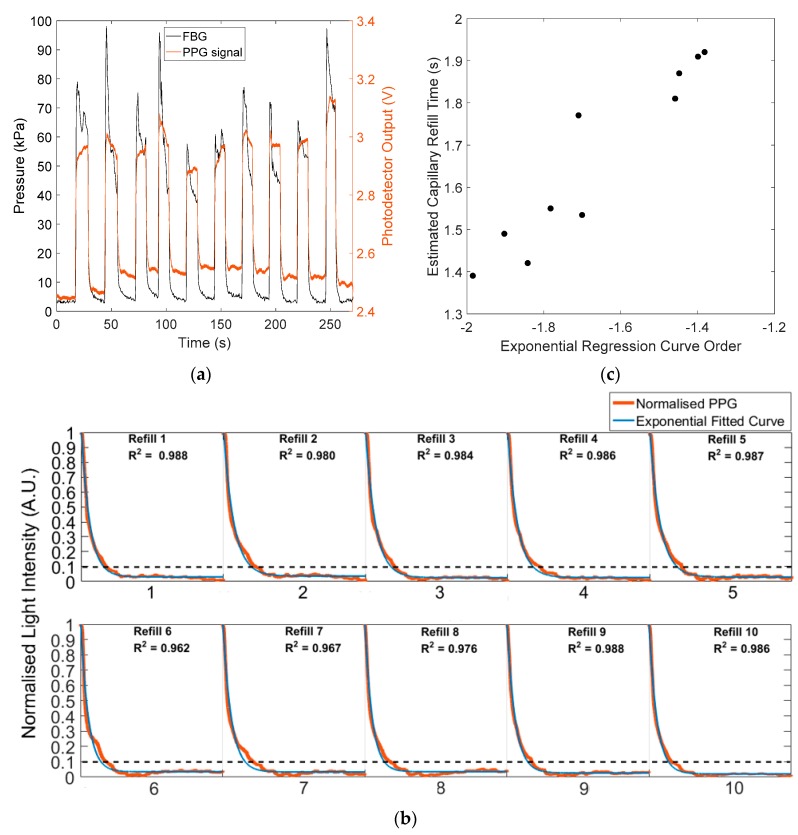
(**a**) Reflected PPG response to blanching pressure. The red line shows the light intensity of the reflected PPG signal, and the black line is the pressure recorded by the FBG. (**b**) Ten normalised capillary refills (red lines) and their exponential regression models (blue lines) over a 10 s period after contact pressure is released. The black dotted line is the 10% threshold applied for estimating CRT. R-squared values for all 10 exponential regression curves are >0.96. (**c**) Estimated CRT (with a threshold level of 10%) versus the order of exponential regression curves.

The new CRT sensor was tested on a group of 10 volunteers using the measurement protocol. Typical results for a single volunteer are presented in Figure 7a to demonstrate the sensor response to blanching pressures. The two signals show the reflected PPG signals (red curves) and the contact pressure (black curve). According to Section 2.3, the refilling intervals are isolated from the reflected PPG signals by extracting the 10-s data after the pressure release. Figure 7b shows 10 normalised capillary refills (red curves) which were isolated from the reflected PPG signal in Figure 7a using the contact pressure record to confirm the pressure release time. The blue curves in Figure 7b are exponential regression curves of isolated and normalised capillary refills. R-squared values of all exponential regression models are >0.96. Figure 7c shows the relationship between the estimated CRT (with a threshold level of 10%) versus the order of exponential model fit. As anticipated, a shorter CRT is characterised by a rapidly decaying exponential fit.

**Figure 8:**
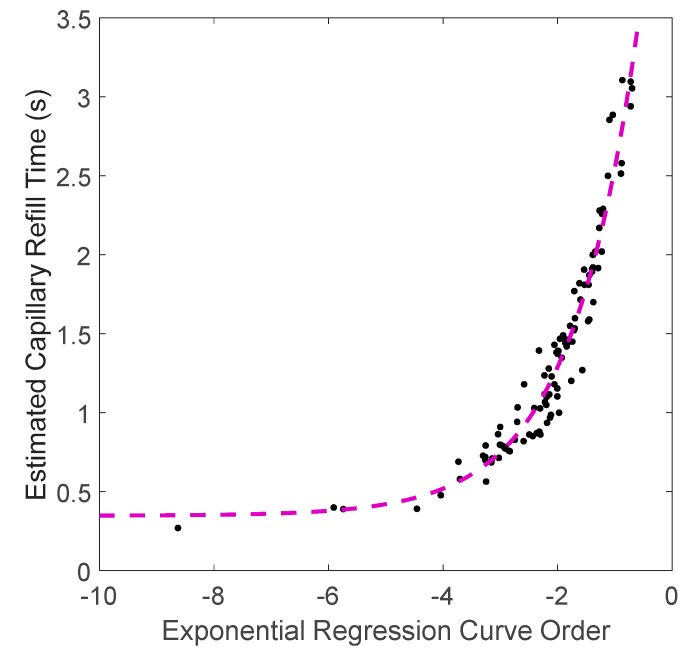
Relationship between the exponential regression curve order and the estimated capillary refill time. The black dots represent the estimated CRT for each refilling process. The purple dashed line is a fit through the data, which indicates an exponential relationship between regression orders and estimated CRT.

The capillary refill responses of all 10 volunteers are shown in the Supplementary Materials. For each of the 10 volunteers, the blanching process was repeated 10 times to provide 100 capillary refill datasets, all of which were similar to Figure 7. Figure 8 shows the relationship between the estimated CRT (with a threshold level of 10%) versus the orders of exponential fitting curves for all 10 volunteers, which indicates an exponential relationship between the regression exponential order (*x*) and estimated CRT (*y*):

(5) $$y=5.16\;*e^{0.85*x}\;+0.35.$$

## 4. Conclusions

In this paper, a novel integrated optical fibre sensor probe combining reflectance photoplethysmography with an FBG contact pressure sensor capable of carrying out measurements of capillary refill time was demonstrated. In vivo measurements of 10 healthy volunteers demonstrated the potential of the sensor to provide quantitative CRT measurements.

The integrated sensor probe has the potential to improve the reliability of CRT measurements. In this case, the applied pressure recorded by the FBG pressure sensor was used to indicate the onset of the release of pressure in order to isolate the capillary refill signal. The CRT signals can be fitted using an exponential regression model, and the order of fit can be related to the CRT. Although previous research has demonstrated that the duration and strength of the blanching pressure can influence the measured CRT, the optimum application pressure and duration are currently poorly standardised [24]. Thus, the probe should find use in optimising the duration and strength of the blanching pressure for CRT measurements. For the data obtained here, we found no clear correlation between the blanching pressure and CRT, which was thought to be important in previous studies [12,13,15]; however, further investigation is required.

In alignment with other PPG measurement systems, motion artefacts will affect signal quality. Some researchers have included an accelerometer to identify motion artefacts [39,40]. However, the FBG pressure sensor provides the capability to identify when contact has been made and when there is relative motion between the sensor and the finger. As described in Section 1, the CRT measurement was also influenced by skin temperature. In this study, the laboratory ambient temperature was maintained in the range of 24 to 26 °C to avoid the effects of skin temperature on CRT measurements. In future studies, calibrating the sensor for skin temperature using either a thermocouple or another FBG temperature sensor located on the surface of the epoxy at the skin’s interface is suggested.

## Supplementary Materials

The following are available online at https://www.mdpi.com/1424-8220/20/5/1388/s1, Figure S1: Temperature performance of FBG 1&2; Figures S2 to S11: 10 groups of experiments data recorded.
