# Supplementary material for: Optical Fibre Sensor for Simultaneous Measurement of Capillary Refill Time and Contact Pressure
Source: Sensors (Basel). 2020 Mar 3;20(5):1388. doi: 10.3390/s20051388 (PMC7085692; doi:10.3390/s20051388)
Supplement: Supplementary file 1 [file sensors-20-01388-s001.pdf]

SUPPLEMENTARY FILE

1) FBG Temperature Compensation

Figure S1 presents the temperature response of FBG1 (pressure sensing FBG) and FBG2 (temperature reference FBG). The Bragg wavelength shift curves of both FBGs follows the same trend in accordance with the temperature changes. In this case, the Bragg wavelength shift of the FBG2 can be directly subtracted from FBG1 to compensate temperature. The empirical equation 1 describes a simple temperature compensation calculation.

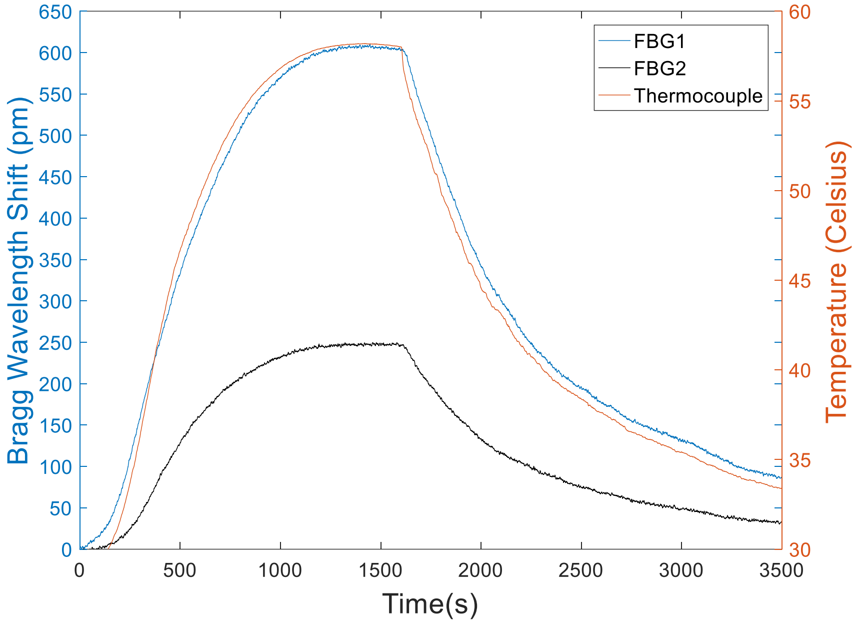

Figure S 1. Temperature performance of FBG 1&2. Blue and black curves are the Bragg (peak) wavelength shift of FBG 1&2 ranging from 30°C to 60°C. The red line presents the output of the themocouple. The peak wavelength of both FBGs shift in accordance with the temperature changes.

2) Experiments Results

In this study, the designed pulse oximetry sensor was applied to measure the reflected light intensity and record the blanching pressure of 10 volunteers during CRT experiments. For each volunteer, the blanching process was repeated ten times. Therefore, there are 10 groups of 100 refilling phase data recorded in total which are shown from Figure S2 to S11. Every figure in the range from S2 to S11 contains 3 diagrams which show the reflected infrared signal (a), the normalised refills with their exponential regression lines (b) and the plot of estimated CRT versus exponential regression curve order (c).

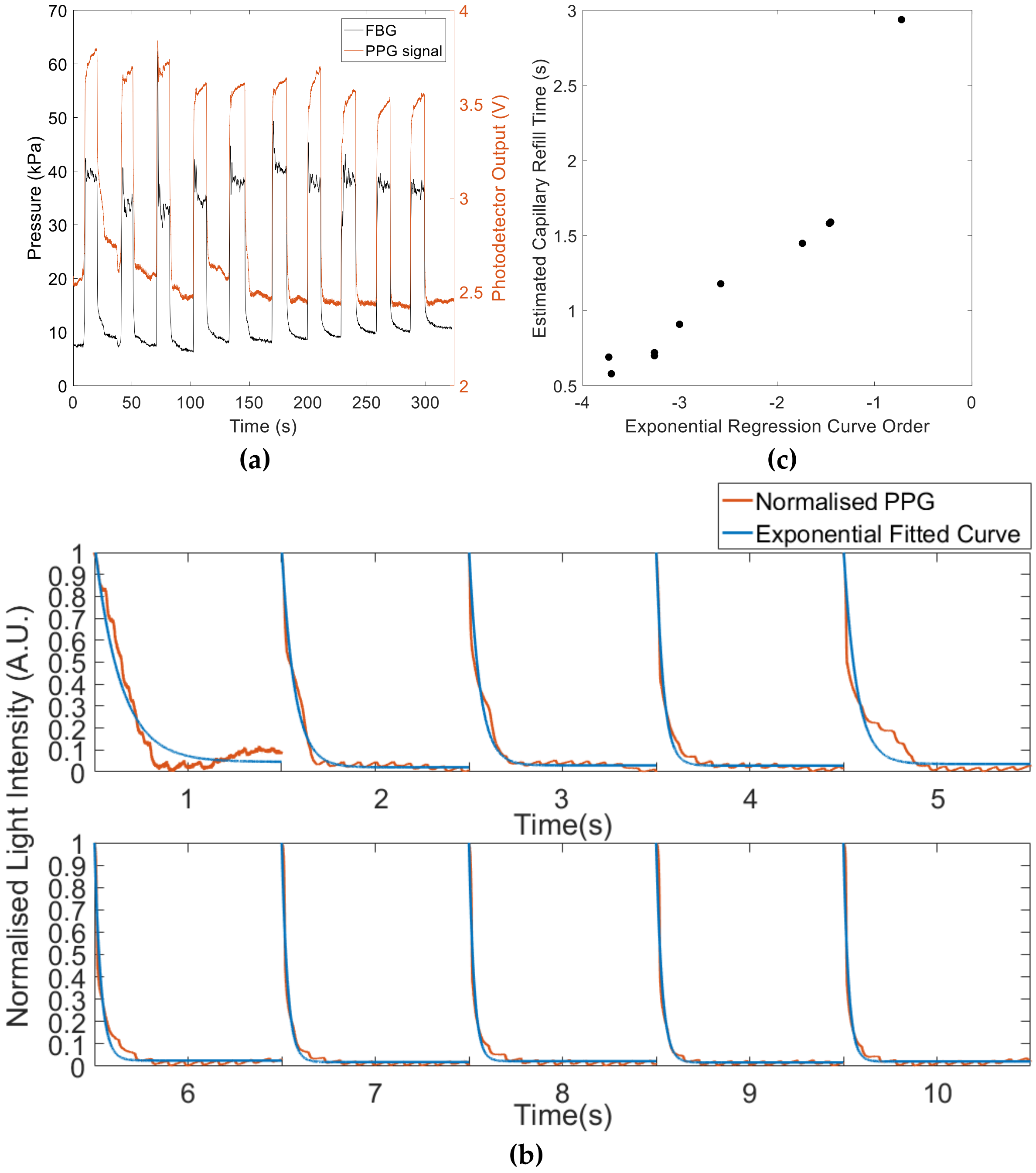

S 2. Volunteer 1. (a) Reflected signal response to the blanching pressure. (b) Ten normalised capillary refills (red lines) and their exponential regression models (blue lines) (c) Estimated CRT versus the order of exponential regression models

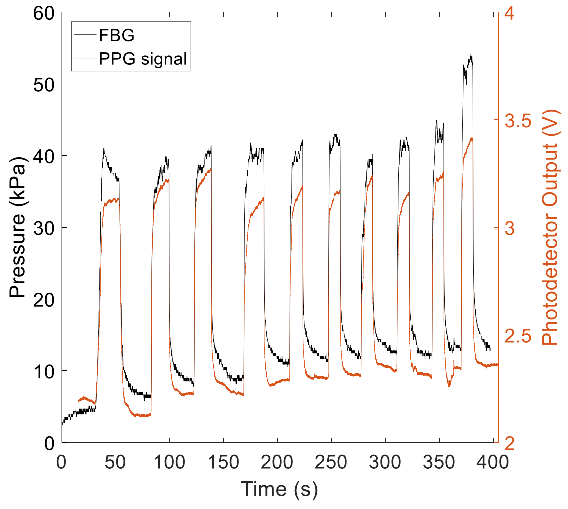

(a)

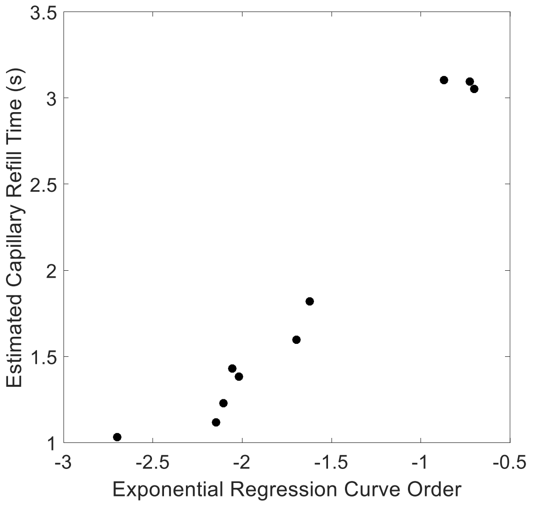

(c)

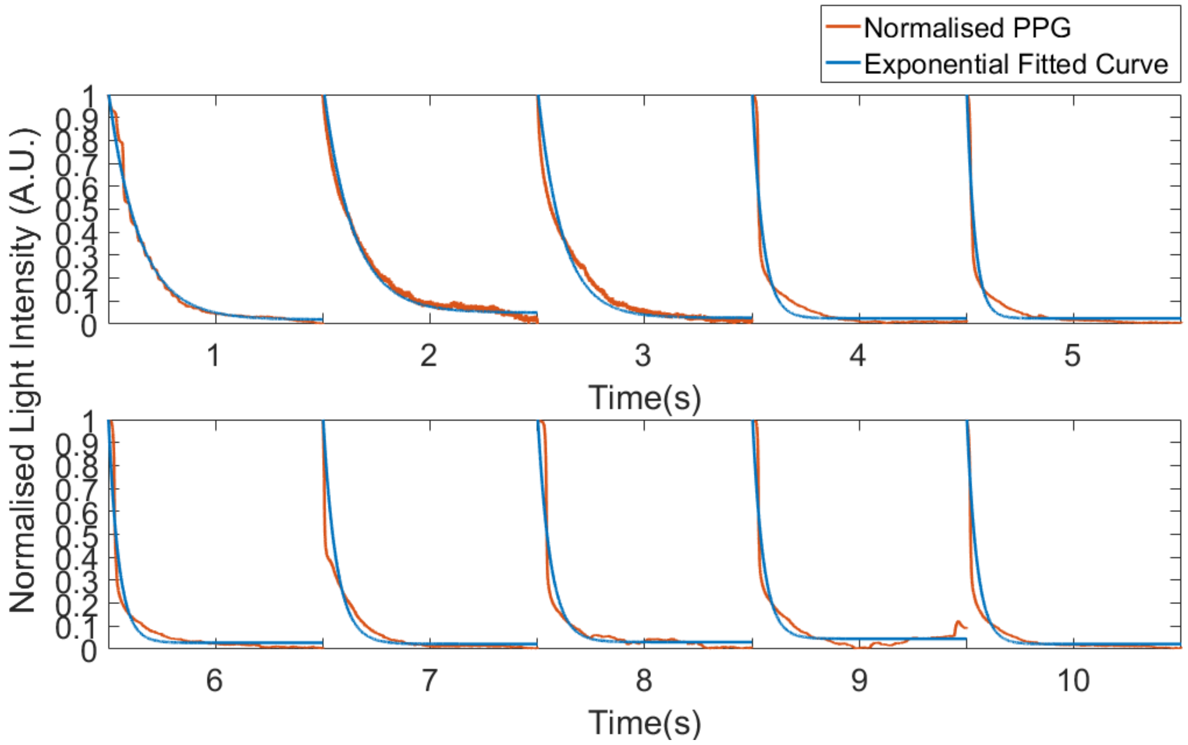

(b)

S 3. Volunteer 2. (a) Reflected signal response to the blanching pressure. (b) Ten normalised capillary refills (red lines) and their exponential regression models (blue lines) (c) Estimated CRT versus the order of exponential regression models

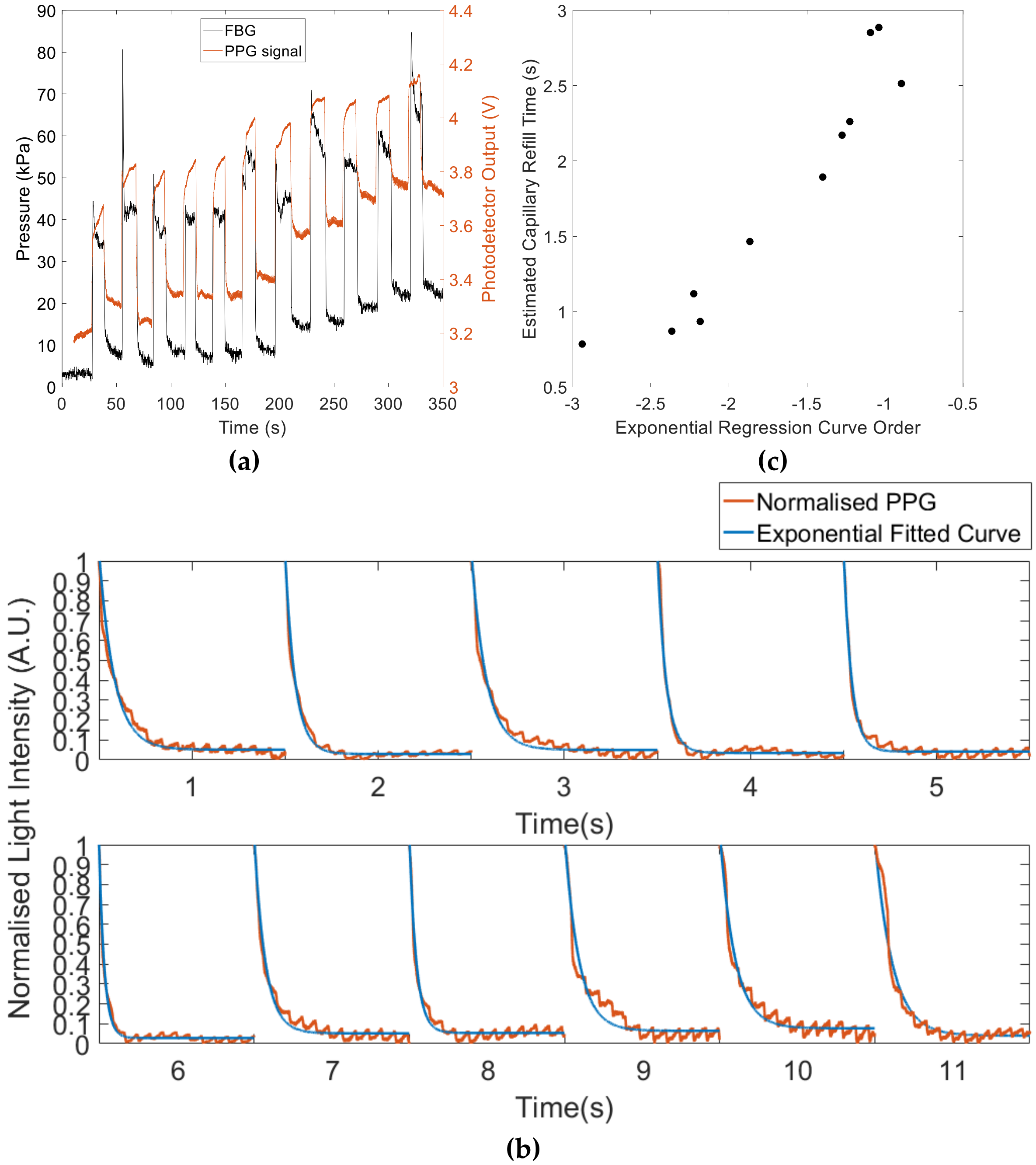

S 4. Volunteer 3. (a) Reflected signal response to the blanching pressure. (b) Eleven normalised capillary refills (red lines) and their exponential regression models (blue lines) (c) Estimated CRT versus the order of exponential regression models

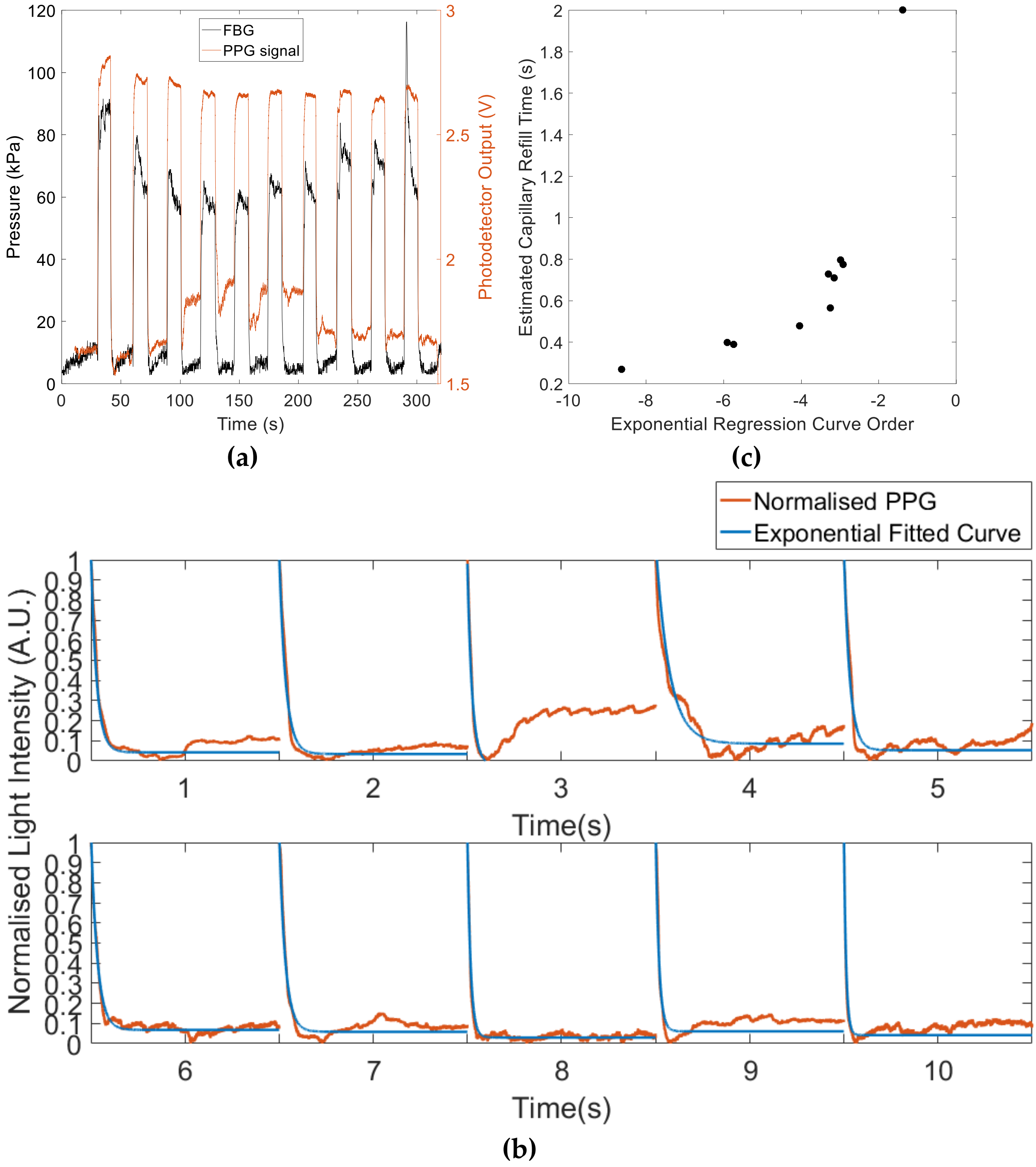

S 5. Volunteer 4. (a) Reflected signal response to the blanching pressure. (b) Eleven normalised capillary refills (red lines) and their exponential regression models (blue lines) (c) Estimated CRT versus the order of exponential regression models

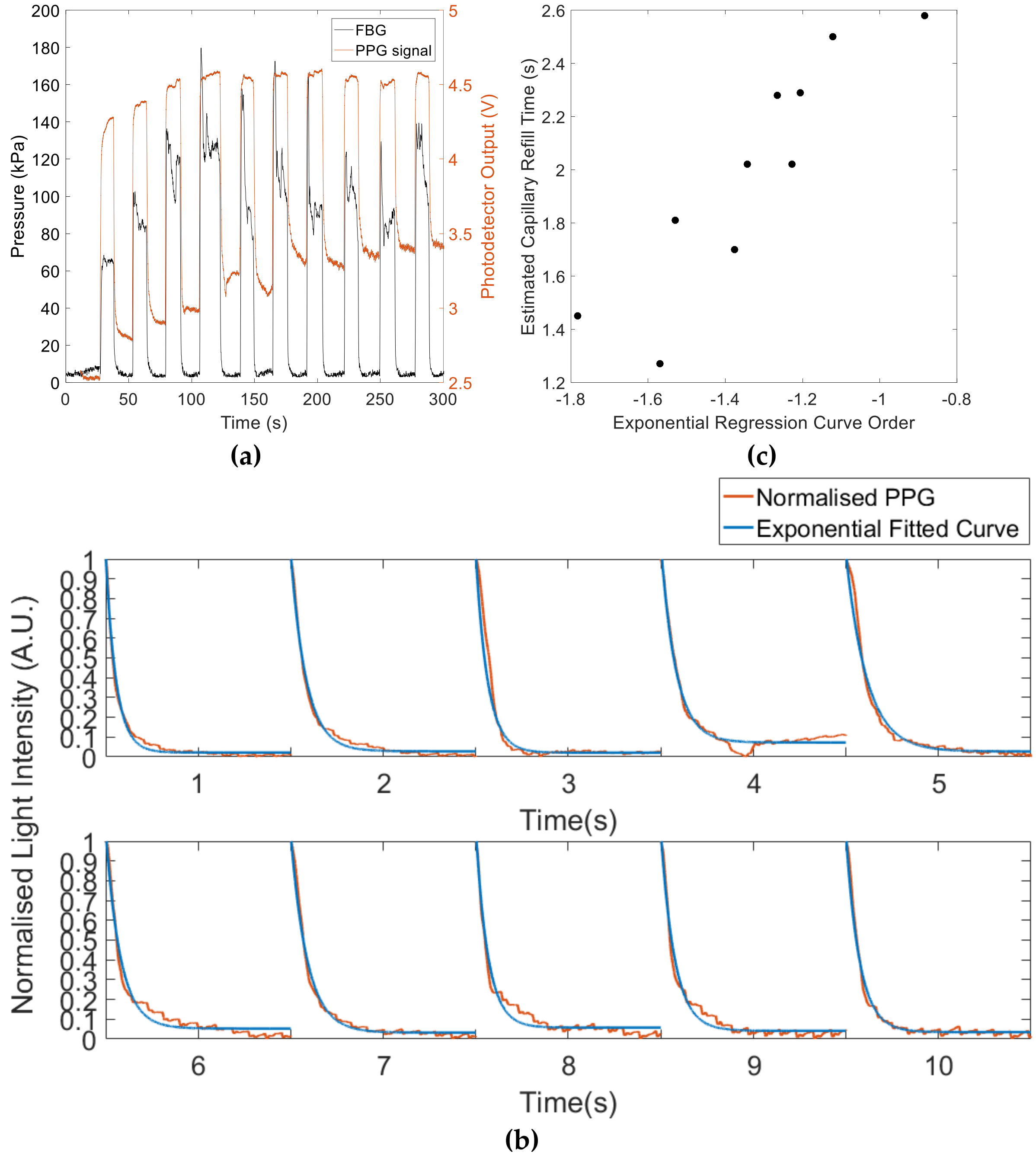

S 6. Volunteer 5. (a) Reflected signal response to the blanching pressure. (b) Ten normalised capillary refills (red lines) and their exponential regression models (blue lines) (c) Estimated CRT versus the order of exponential regression models

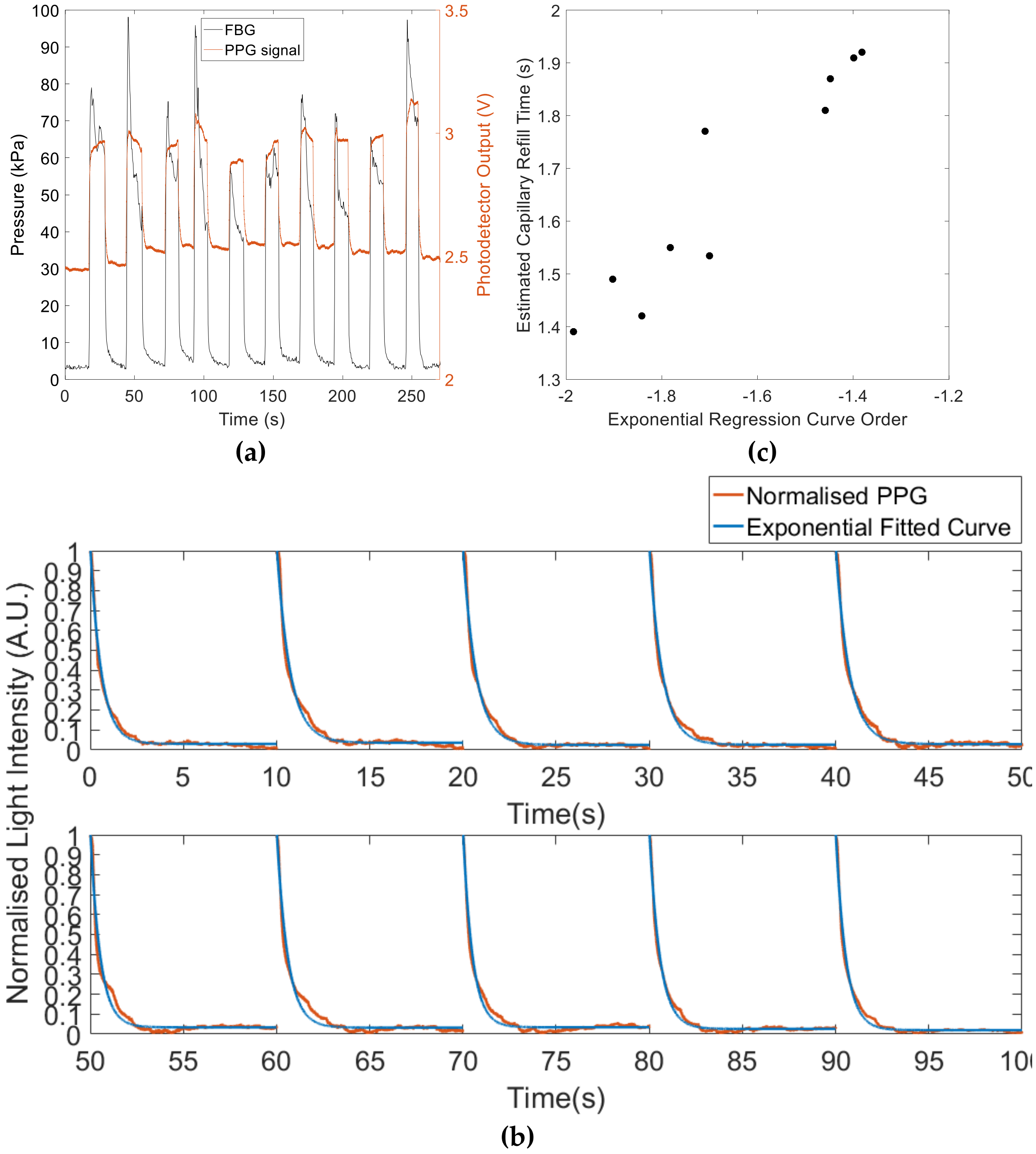

S 7. Volunteer 6. (a) Reflected signal response to the blanching pressure. (b) Ten normalised capillary refills (red lines) and their exponential regression models (blue lines) (c) Estimated CRT versus the order of exponential regression models

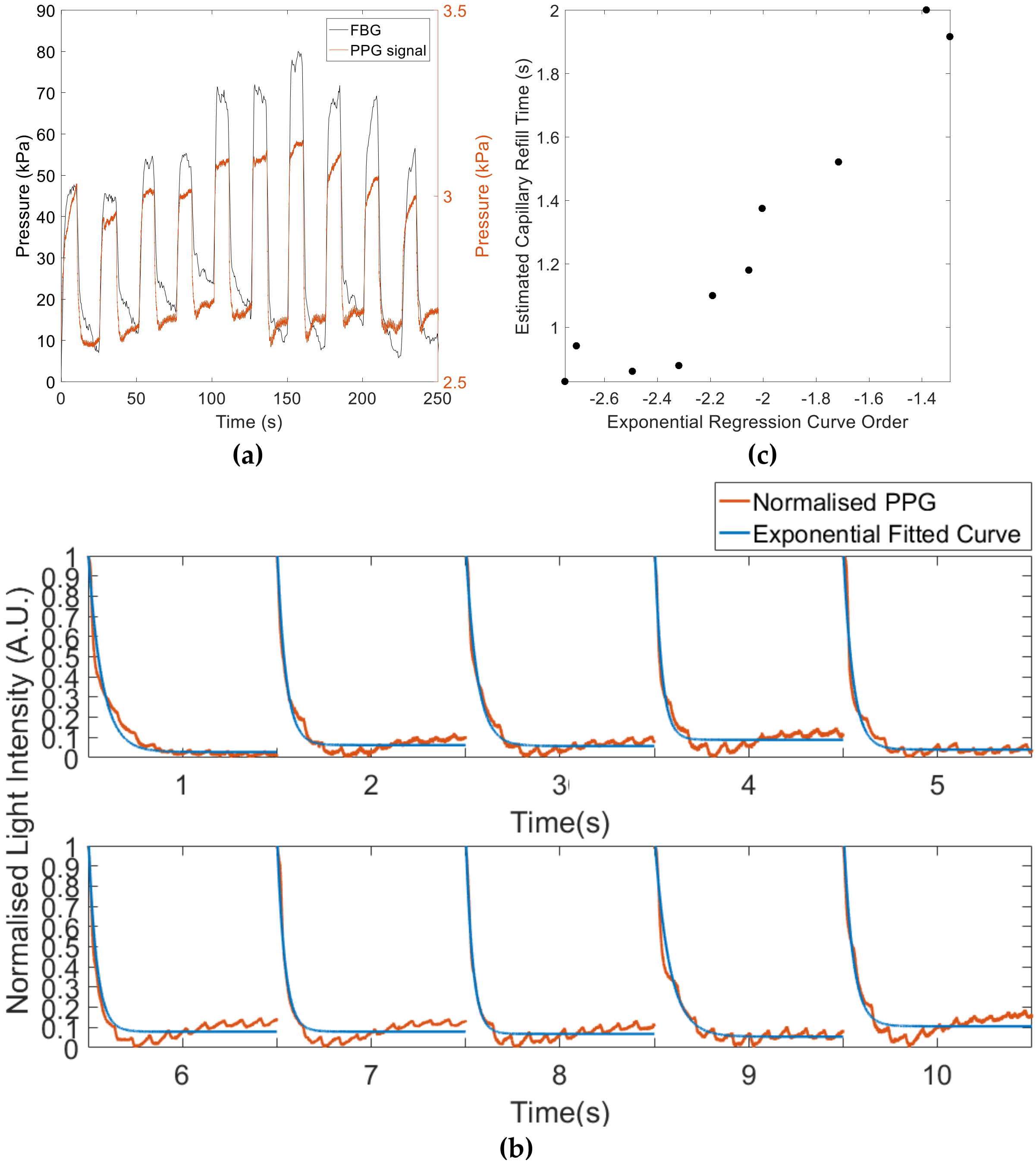

S 8. Volunteer 7. (a) Reflected signal response to the blanching pressure. (b) Ten normalised capillary refills (red lines) and their exponential regression models (blue lines) (c) Estimated CRT versus the order of exponential regression models

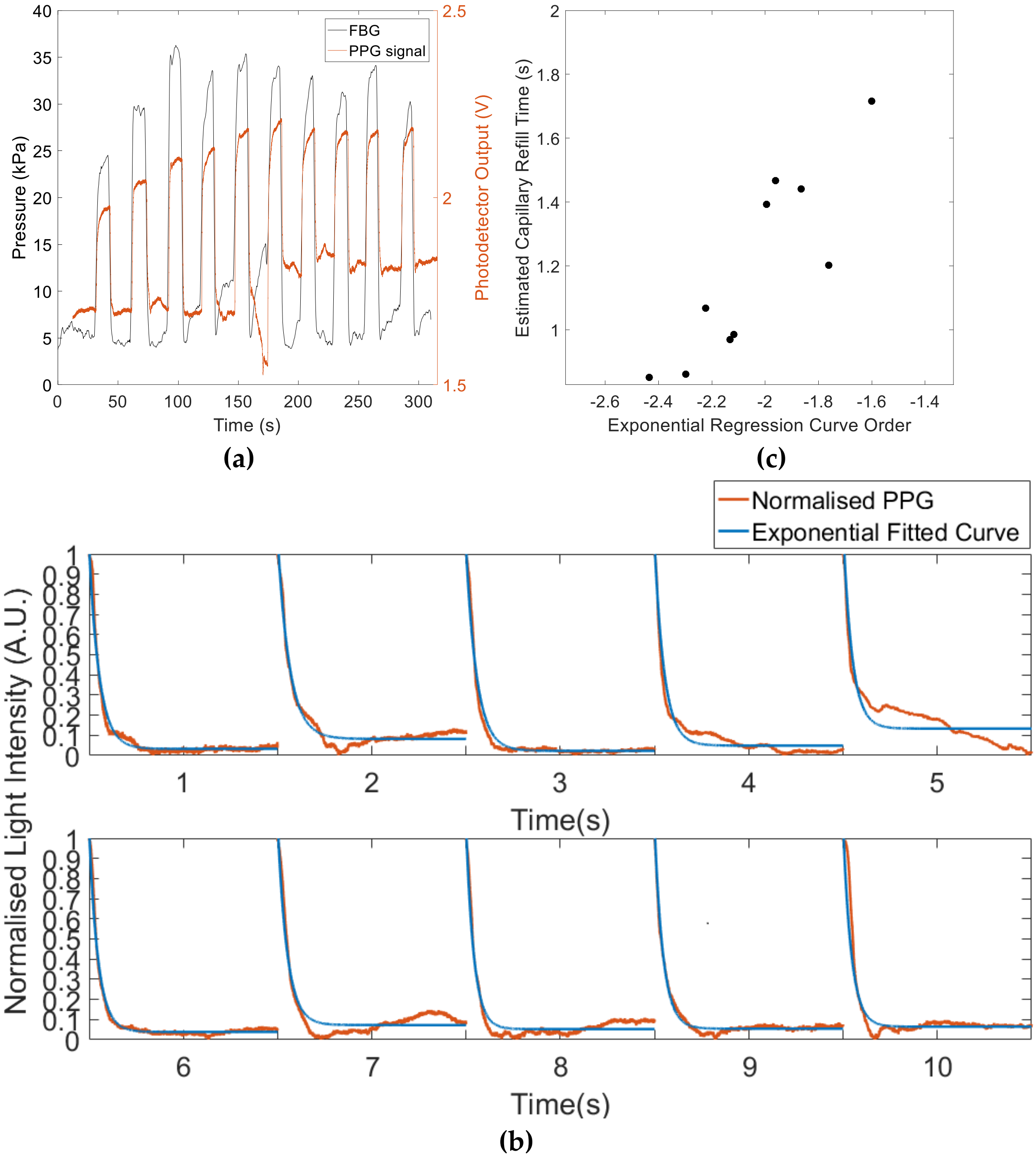

S 9. Volunteer 8. (a) Reflected signal response to the blanching pressure. (b) Ten normalised capillary refills (red lines) and their exponential regression models (blue lines) (c) Estimated CRT versus the order of exponential regression models

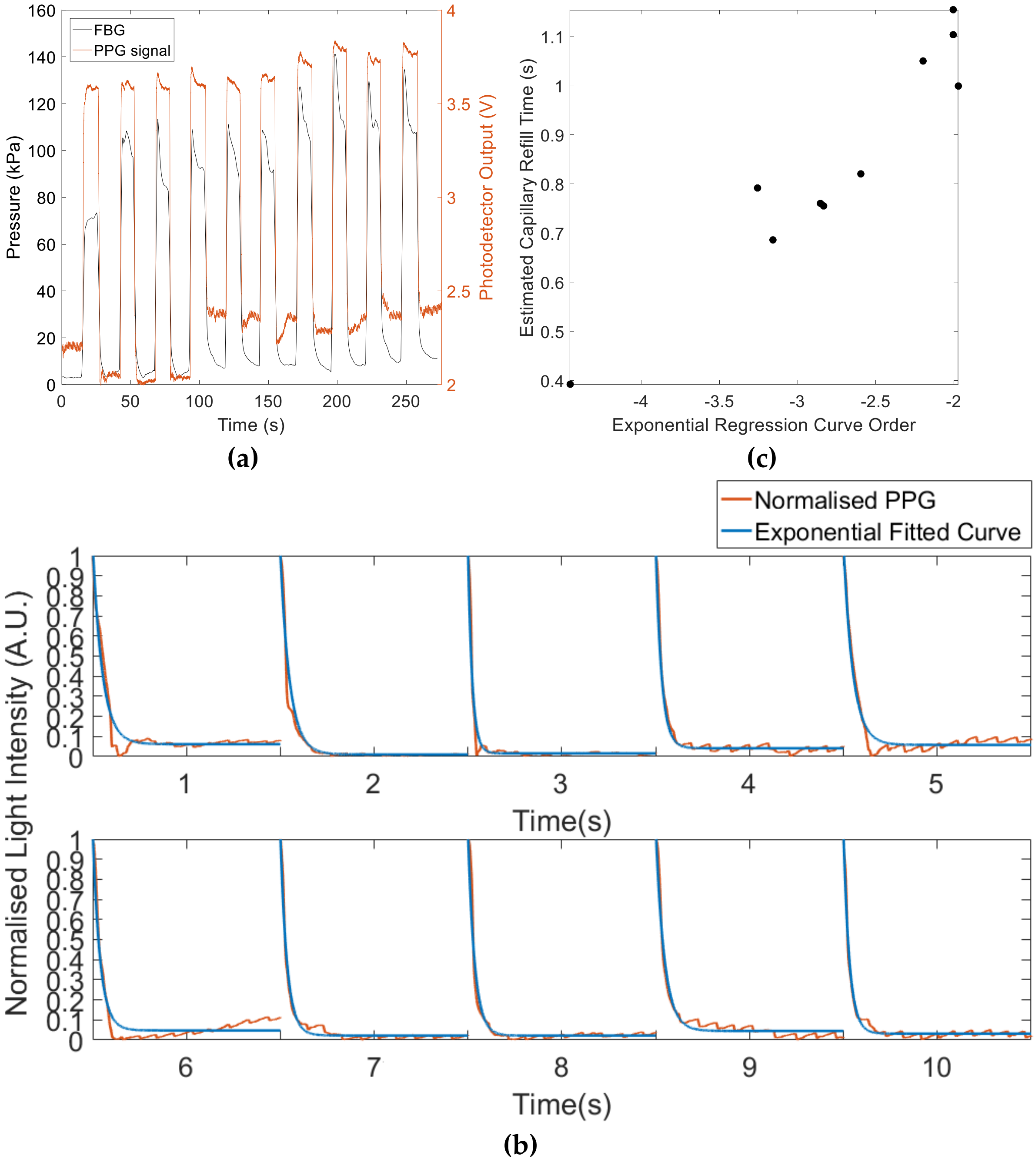

S 10. Volunteer 9. (a) Reflected signal response to the blanching pressure. (b) Ten normalised capillary refills (red lines) and their exponential regression models (blue lines) (c) Estimated CRT versus the order of exponential regression models

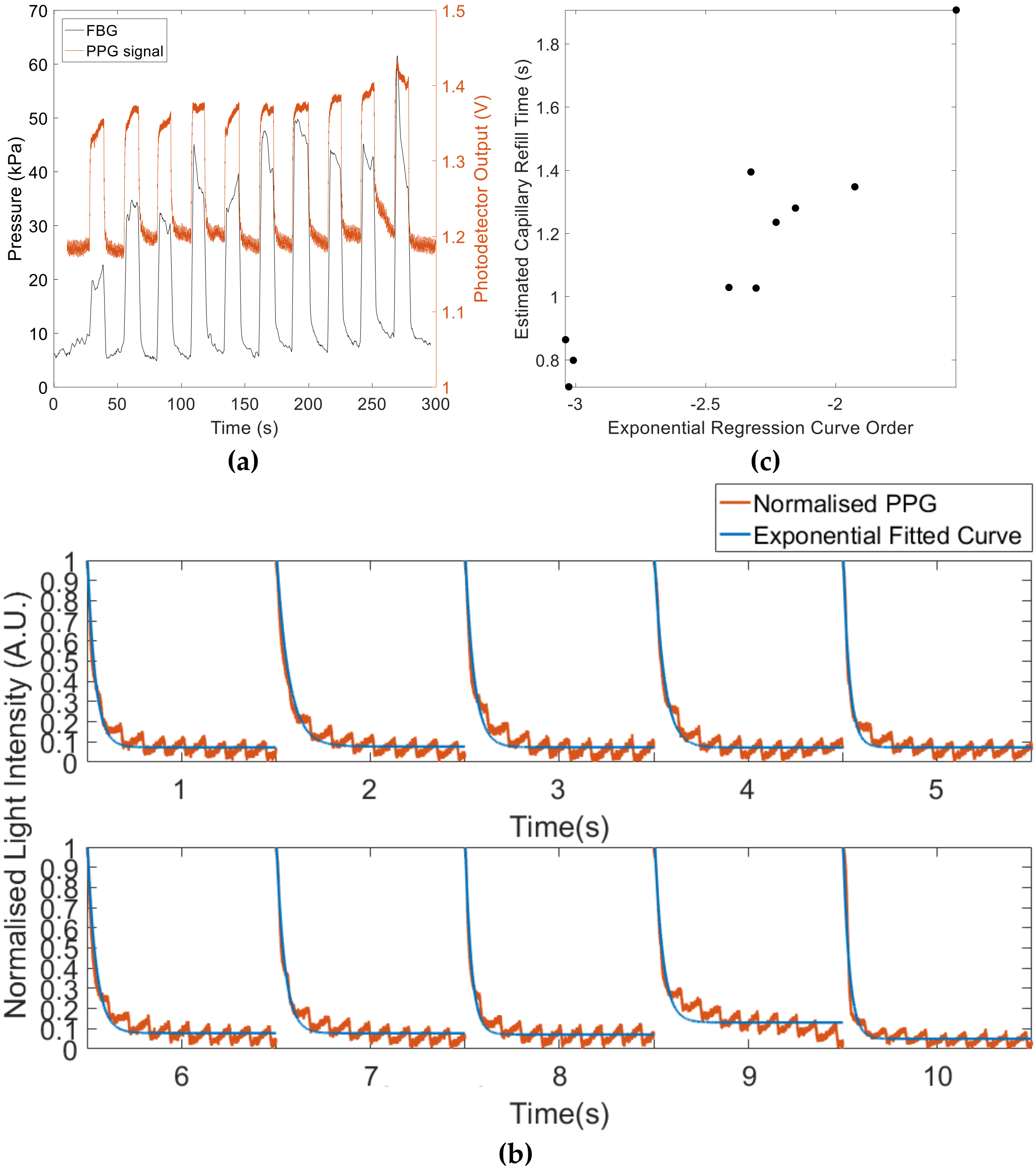

S 11. Volunteer 10. (a) Reflected signal response to the blanching pressure. (b) Ten normalised capillary refills (red lines) and their exponential regression models (blue lines) (c) Estimated CRT versus the order of exponential regression models
